# Zinc Deficiency, Plasma Fatty Acid Profile and Desaturase Activities in Hemodialysis Patients: Is Supplementation Necessary?

**DOI:** 10.3389/fnut.2021.700450

**Published:** 2021-09-23

**Authors:** Marija Takic, Milica Zekovic, Brankica Terzic, Aleksandar Stojsavljevic, Mirjana Mijuskovic, Slavica Radjen, Danijela Ristic-Medic

**Affiliations:** ^1^Centre of Research Excellence in Nutrition and Metabolism, Institute for Medical Research, University of Belgrade, Belgrade, Serbia; ^2^Clinic of Nephrology, Military Medical Academy, Belgrade, Serbia; ^3^Medical Faculty of the Military Medical Academy, University of Defence in Belgrade, Belgrade, Serbia; ^4^Innovation Center of the Faculty of Chemistry, Department of Analytical Chemistry, University of Belgrade, Belgrade, Serbia; ^5^Institute of Hygiene, Military Medical Academy, Belgrade, Serbia; ^6^Department of Nutrition Biochemistry and Dietology, Institute for Medical Research, University of Belgrade, Belgrade, Serbia

**Keywords:** zinc, fatty acid, Cu/Zn ratio, LA/DGLA, hemodialysis

## Abstract

**Background:** Desaturation and elongation are critical processes in endogenous metabolic fatty acid pathways. Zinc (Zn) is a cofactor for desaturases and elongases enzymes. There is limited evidence regarding the relationships between biomarkers of Zn status, nutritional intake, plasma phospholipid fatty acid profile and clinical outcomes among patients undergoing hemodialysis (HD).

**Objective:** To examine the relationships between dietary and serum levels of Zn and Cu/Zn ratio and to explore associations of these micronutrients with PUFA profile and estimated desaturase and elongase enzyme activities in serum phospholipids among HD patients.

**Methods:** This study included 40 adult patients undergoing hemodialysis treatment. Repeated 24-h recalls were applied for dietary intake assessment. Serum concentration of Zn and Cu were determined using inductively coupled plasma mass spectrometry and fatty acid composition by gas-liquid chromatography. Desaturase and elongase activities were calculated from product-precursor fatty acid ratios.

**Results:** Inadequate dietary Zn intake was found in 55% of HD patients. They all had serum Zn concentration below the reference value of 60 μg/dL (mean 38.8 ± 7.72 μg/dL). Adequate zinc intake was accompanied with significantly higher intake of energy, total fats, SFA, MUFA and proteins. There was no correlation between Zn serum status and Zn intake estimates. Serum Cu/Zn ratio was high, (2.76 ± 0.68), directly and significantly associated with HD period, CRP, BMI, VFA, and inversely with Kt/V, albumin, iron, and iPTH. The n-6/n-3 ratio in plasma phospholipids was elevated (12.25 ± 3.45) and patients with inadequate Zn intake had lower n-3 PUFA intake and status compared to those with adequate intake. Serum Zn concentrations were inversely correlated with linoleic/dihomo-γ-linolenic acid ratio (LA/DGLA) (*p* = 0.037), related to D6-desaturase activity (*p* = 0.033) and directly with DGLA relative abundances (*p* = 0.024). Cu status was inversely associated with EPA level (*p* = 0.03) and estimates of elongase activity (*p* = 0.001). Furthermore, positive relationship was found between the Cu/Zn ratio and determined elongase value (*p* = 0.01).

**Conclusion:** Findings of this study underpin the high prevalence of Zn deficiency and inadequate n-3 PUFA intake and status among subjects undergoing HD. The results obtained indicate that the assessment of Zn status should be a standard parameter of nutritional status screening in HD patients while emphasizing the importance of Cu/Zn determination. Although further research is warranted, Zn and-n-3 PUFA supplementation in HD patients might be beneficial for the prevention and attenuation of adverse health outcomes

## Introduction

Chronic kidney diseases (CKD) is recognized as a global public health concern with significant clinical, economic, and humanistic burdens. Patients on hemodialysis treatment have a high incidence of cardiovascular diseases (CVD) and an increased mortality rate (1). Risk factors such as dyslipidemia, insulin resistance, disturbances in fatty acid metabolism, increased oxidative stress, and inflammation are considered accountable for the endothelial impairment and cumulative alterations of vascular function in hemodialysis patients (2). Zinc is an essential micronutrient with numerous roles is fundamental biologic processes. Due to unique antioxidant and anti-inflammatory properties and implications in enzyme activity, membrane stabilization and apoptosis inhibition, zinc is essential for endothelial integrity (3). Consequently, zinc deficiency could lead to severe endothelial damage and amplification of the detrimental impact of certain fatty acids such as linoleic acid, and inflammatory cytokines on vascular function (4). There is accumulating evidence that dysregulation of zinc metabolism and suboptimal status could be associated with the development of CVD (5). The risk of atherosclerosis in zinc-deficient patients with renal failure plays an important role in the progression and complications of CKD (6, 7). Lower zinc levels are related to end-stage renal disease (8).

Zinc deficiency increases oxidative stress and a cytokine-mediated inflammatory process leading to atherosclerotic complications (3). Among patients with CKD, deterioration of kidney function causes impaired elimination of an array of uremic toxins. They have been shown to promote inflammatory state and aggravate the production of reactive oxygen species. The damaging effect of these processes is intensified by the hemodialysis procedure itself, and, in such circumstances, zinc exerts a significant role in antioxidant systems. Numerous studies underpinned Cu/Zn-superoxide dismutase gradient as an effective oxidative stress marker for the evaluation of progressive renal damage (8). It has been proposed that the serum Cu/Zn ratio reflects the reciprocal reaction of Cu and Zn better than serum Cu or Zn concentrations separately (9, 10). Furthermore, it was found that the Cu/Zn-ratio significantly increases with aging (11). This biomarker, used also for the evaluation of oxidative stress burden, is associated with a higher risk of incident infection (10) and elevated cardiovascular mortality rate in the elderly population (12–14). Zinc, as an antioxidant and anti-inflammatory mediator, regulates the function of lymphocytes, making the body's immune system less susceptible to various infections (15–17). Recently, the protective role of zinc in vulnerable groups for COVID-19 such as hemodialysis patients has been mentioned (18).

Several studies confirm a high prevalence of zinc deficiency in hemodialysis patients (5, 19–23). This could be caused by multiple factors such as: inadequate nutritional intake due to protein restriction, reduced gastrointestinal dietary absorption, increased zinc excretion or loss into the dialysate, altered cellular and tissue distribution, as well as phosphate-binder drugs interaction and oral iron supplementation (16, 24–26). The main inhibitor of intestinal zinc absorption is phytate, which is found in unrefined cereals, pulses, oilseeds, and nuts. The evidence indicates that zinc deficiency could lead to erythropoietin-resistant anemia and increased susceptibility to renal damage in patients with diabetes (27, 28).

Previous studies, including ours, have reported disturbances in the proportion of serum fatty acids in hemodialysis patients particularly with lower n-3 polyunsaturated fatty acids (PUFA) and higher monounsaturated fatty acids (MUFA) content compared to healthy subjects (29–32). Depending on stages of CKD severity, there is an increase of MUFA and a gradual progressive decrease in the content of n-3 and n-6 PUFA, which contributes to the occurrence of dyslipidemia in CKD patients (33). The mechanism by which disturbance in fatty acid profile such as decline of n-3 PUFA/arachidonic acid (AA) ratio in hemodialysis still remains unclear (34). Endogenous fatty acid metabolism is regulated by D5, D6, and D9 desaturase enzymes. Zinc is a cofactor for desaturases and elongases enzymes (35). Therefore, the alterations of zinc serum levels could affect the activities of these enzymes and consequently modulate the regulation of fatty acid metabolism. Recently, the linoleic (LA)/ dihomo-γ-linoleic acid (DGLA) ratio has been proposed as a novel biomarker of zinc status (36, 37). There is a scarcity in available scientific data regarding the interaction between zinc intake and status and PUFA fatty acid in CKD.

Thus, the aim of the present study was to examine the relationship between dietary zinc intake, its serum concentrations and Cu/Zn ratio and to explore associations of these micronutrients with PUFA profile in serum phospholipids and estimated desaturase and elongase enzyme activities in hemodialysis patients.

## Materials and Methods

### Study Design

This was a cross-sectional study conducted at the Department of Hemodialysis, Clinic of Nephrology, Military Medical Academy, Belgrade, Serbia.

### Study Population

We studied 40 patients undergoing hemodialysis treatments between February 2017 and January 2019. All patients were dialyzed three times per week for 4 h with high-permeability membranes. The blood flow was in the range of 250–300 mL/min with a dialysis rate flow of 500 mL/min. Oral iron supplementation, based on ferrous citrate or an iron containing phosphate binder was administered to all the participants. The exclusion criteria were heart failure (NYHA III or IV), acute myocardial infarction, and acute infectious disorders within three months of recruitment. Patients were not obese and had no severe malnutrition (BMI 20–30 kg/m^2^). After the initial eligibility screening, subjects consuming zinc-salts and omega 3 dietary supplements were excluded. Involved patients voluntarily provided the written informed consent to participate in this research. This study was conducted in accordance with standards and a principle stated in the Declaration of Helsinki and was approved by the Ethical Review Board of the Military Medical Academy, Belgrade, Serbia (Approval Project No 8/15-17).

### Analysis of Biochemical Parameters

All blood samples were obtained immediately before the mid-week dialysis session after a 12-hour fast. The following serum parameters were considered: urea, creatinine, and potassium, C-reactive protein (CRP), hemoglobin (Hb), total cholesterol, HDL- and LDL-cholesterol, triglycerides, albumin, iron, vitamin D, and intact parathyroid hormone (iPTH). After the dialysis procedure serum urea, creatinine and potassium analyses were repeated. Concentrations of biochemical blood parameters were obtained spectrophotometrically using the Siemens Dimension Rxl Max analyzer. The value of CRP was calculated *via* enhanced turbidimetric-immunoassay (PETIA) using the Siemens Dimension RxlMax analyzer. The Kt/V value quantifying the hemodialysis efficiency was calculated using the formula proposed by Daugirdas and Blake (38). Aliquots of the remaining serum were made immediately, and these samples were stored at −80 °C for further analysis of trace metal profiles (zinc, copper) and fatty acids.

### Anthropometric Measurements

Assessment of anthropometric parameters included height, weight, mid-arm circumference (MAC) and waist circumference (WC). Measurement was performed with individuals wearing light clothes, without shoes. Height was measured using wall-mounted stadiometer to the nearest 0.5 cm. Body weight and % of body fat was measured using a body composition analyzer InBody720 (Biospace Co., Ltd., Seoul, Korea). Visceral fat area (VFA) was measured at the level of the umbilicus with a dual bioelectrical impedance analyzer (DUALSCAN, Omron Healthcare Co, Kyoto, Japan). WC was measured from the midpoint between the lateral iliac crest and the lowest rib to the nearest 0.5 cm. The visceral adiposity index (VAI) was calculated using the formula by Amato et al. (39).

### Assessment of Dietary PUFA, Zinc and Copper Intake

Dietary intake evaluation was based on participants' subjective retrospective report, using the repeated 24-h recalls as the assessment method. Complete consumption of food and beverages was recorded on three occasions (dialyses day, one non-dialyses weekday and one non-dialyses weekend day) for each individual. Data was collected within in-depth multiple-pass direct structured interviews administered by skilled survey staff. Quantities of food consumed were estimated in reference to common household measures, natural units, and standard measuring kitchen tools as well as container size or packaging information for commercial products. Regionally specific, previously validated Food Atlas containing 135-items (simple foods and composite dishes) was applied as an additional two-dimensional portion-size estimation aid (40). Questionnaires were processed and analyzed *via* Diet Assess & Plan, an advanced nutritional software tool (41). Conversion of food to nutrient intake was performed based on mean values for three recall replicates and all the listed food items were matched and coded using the national Serbian Food Composition Database (42) in the Institute for Medical Research. Estimates of zinc intake were compared against reference values proposed specifically for hemodialysis patients, i.e.10–15 mg for men and 8–12 mg for women (43, 44). Adequacy assessment for copper intake was based on European Food Safety Authority (EFSA) recommendations for general population (1.3 mg/day for women, 1.6 mg/day for men) (45).

### Fatty Acid Determination and Estimation of Desaturase Activity

Total serum lipids were extracted according to the method of Folch (46). As previously described, plasma phospholipids were isolated by one-dimensional thin-layer chromatography (TLC) in neutral solvent system hexane-diethyl-ether acetic acid (87:12:1 v/v) using Silica Gel GF plates (C. Merck, Darmstadt, Germany) and trans esterified to methyl fatty acid (47). Afterwards, phospholipids' fatty acid methyl ester samples were analyzed by gas–liquid chromatography on the Shimadzu chromatograph GC 2014 (Kyoto, Japan), equipped with a flame ionization detector on an Rt × 2330 column (60 m × 0.25 mm ID, film thickness of 0.2 μm; RESTEK, Bellefonte, PA, USA) with initial oven temperature 130 °C held for 10 min and then programmed to increase at 3 °C/min to a final oven temperature of 220 °C. Fatty acid methyl esters were identified by comparing peak retention times with retention times obtained for fatty acids components of standard mixtures PUFA−2 and/or 37 FAMEs mix (Supelco, Bellefonte, PA, USA). The relative abundances of individual fatty acids were calculated and expressed as a percentage of total identified FAs. Activities of fatty acid desaturases (D) were estimated as ratios of their relative abundances as follows: 18:1n-9/18:0 for stearoil-CoA D18(SCD-18), 16:1n-7/16:0 stearoil-CoA D16 (SCD-16), 20:3n-6/18:2n-6 for delta-6D (D6D), 20:4n-6/20:3n-6 delta- (D5D) and 22:5n-3/20:5n-3 for elongase (ELO) (48).

### Serum Zinc and Copper Determination

Serum samples were prepared according to Stojsavljević et al. (49). All chemicals used were of high grade and were supplied by Merck (Darmstadt, Germany). Briefly, all samples for metal analysis were diluted ten times with an aqueous solution containing 0.05% nitric acid, 0.1% Triton X-100, and 3% 1-butanol. Copper and zinc were determined by inductively coupled plasma-mass spectrometry (ICP-MS) in the collision mode with internal standardization. Linearity of the calibration curve above 0.999 was obtained for both trace metals in the range from 1 to 250μg/L. The accuracy of the method used, expressed as recovery and determined by analysis of standard reference material (Seronorm™ Trace Elements Serum L-2), was from 95.2 to 103.3% for ^65^Cu and from 96.2 to 102.7% for ^66^Zn. Serum zinc concentration below 60 μg/dL was defined as zinc deficient (50).

### Statistical Analysis

Complete Statistical analysis was performed with IBM SPSS 23 software package (Chicago, IL, USA) and principal component analysis (PCA) in PLS ToolBox, v.6.2.1, Matlab 7.12.0 (R2011a) statistical package. The distribution of data was evaluated using Shapiro-Wilk's test and Grubbs' test-a and principal PCA to test outliers. Performed test showed that there were no outliers. The results obtained were presented as the mean value ± standard deviation (SD) for normally distributed, and medians (interquartile range) for scattered variables. Mean values of normally distributed data were compared by unpaired Student's *t*-test and non-parametric Mann-Whitney test was performed on non-transformed scattered values data. A logarithmic transformation was performed for scattered variables prior to further analyses with Pearson's/Spearmen's test for correlation analysis. For all the analyses a *p*-value < 0.05 was considered indicative for statistical significance.

## Results

### Dietary Zinc, Copper and PUFA Intake in Hemodialysis Patients

**Figure 1:**
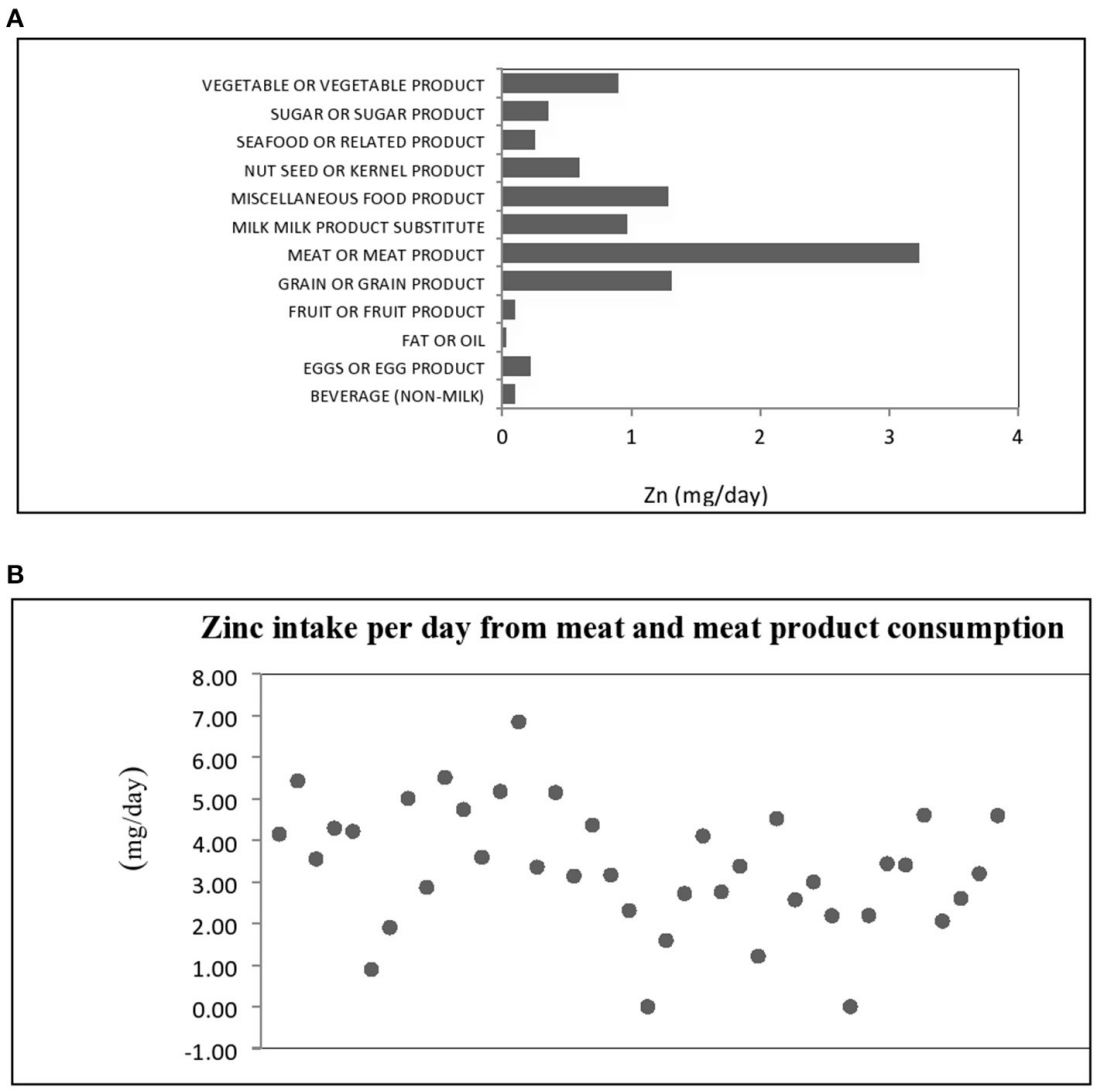
**(A)** Dietary sources of zinc intake by food groups and **(B)** Distribution of zinc intake for meat and meat product in hemodialysis patients.

Forty participants with a mean age of 56 ± 15 years were enrolled in the present study. The sample comprised thirty-one (78%) male and nine female (22%) hemodialysis (HD) patients. As it is shown in Table 1, 22 (55%) participants had inadequate dietary zinc intake compared to recommended values for HD patients (10–15 mg/day for men, 8–12 mg/day for women). Distribution of food groups of zinc dietary sources among study participants, based on 24 h recalls, is presented in Figure 1. Biomarker analyses revealed that all HD patients had zinc deficiency (i.e. serum zinc concentration below the 60 μg/dL threshold) with a mean value of 38.8 ± 7.8 μg/dL. Accordingly, patients' subdivision into categories based on zinc status was not possible. With a median value of 629 mg/day, copper intake was significantly below the recommended level for the general population (45). Serum copper concentration and the Cu/Zn ratio were 97.3 μg/dL and 2.8, respectively (Table 1).

**Table 1:** Demographic characteristic, dietary intake data, zinc and copper status in hemodialysis patients.

**Variable**	**All patients**	**Adequate Zn intake**	**Inadequate Zn intake**
Age (years)	56 ± 15	56 ± 14	53 ± 15
Gender (M/F) n (%)	31/9 (78/22%)	15/3	16/6
BMI (kg/m^2^)	24.49 ± 3.76	25.09 ± 4.12	24.62 ± 3.44
HD duration (years)	5 (2–11.5)	3.5 (2–5)	5 (2–11)
Smokers/non-smokers n (%)	17/23 (42/58%)	9/9	8/14
**Dietary zinc intake (mg/day)**	9.35 ± 3.38	12.13 ± 2.56	7.07 ± 1.95^***^
M (RDI in HD 10-15 mg/day)	12.63 ± 2.49		
F (RDI in HD 8-12 mg/day)	9.63 ± 2.10		
M <10 mg/day n (%)	16/31 (52%)		
F <8 mg/day n (%)	6/9 (67%)		
**Dietary copper intake (μg/day)**	629 (425–774)	664 (512–799)	525 (402–749)
Energy intake (kcal/day)	1,858 (1,687–2,177)	2,093 ± 420	1822 ± 232^*^
Protein (g/day)	72.0 (59.0–87.7)	87.7 (78.9– 99.8)	63.6 (55.8–72.8)^**^
Carbohydrate (g/day)	230 ± 45	233 ± 62	228 ± 26
Total fiber (g/day)	19.5± 5.9	19.18 ± 5.33	19.77 ± 6.44
Fats (g/day)	79.8 ± 24.1	89.1 ± 18.5	70.9 ± 22.9^**^
SFA (g/day)	30.77 ± 12.04	38.42 ± 10.71	27.72 ± 11.25^**^
MUFA (g/day)	24.84± 7.57	31.06 ± 6.67	23.82 ± 9.11^*^
**Dietary PUFA intake (g/day)**	15.53 (8.72–20.00)	16.60 (9.07–19.96)	15.20 (7.18–26.02)
n−3 PUFA (g/day)	0.581 (0.507–0.836)	0.687 (0.560–0.942)	0.528 (0.394–0.688)^*^
n-6 PUFA (g/day)	12.60 ± 6.88	12.85 ± 5.96	12.39 ± 7.68
n-6/n-3	17.86 (10.16–29.00)	19.12 (10.20–27.07)	16.70 (10.12–36.72)
**Serum**			
Zinc (μg/dL)	38.8 ± 7.72	35.2 ± 5.4	39.8 ± 9.3
Serum zinc deficiency			
(<60 μg/dL) n (%)	40 (100 %)		
Correlation: Zn intake/status	*r* = −0.200		
	*p* = 0.211		
Copper (μg/dL)	973 (930–1,191)	1,037 (719–1,108)	980 (930–1,086)
Cu/Zn ratio	2.76 ± 0.68	2.85 ± 0.68	2.56 ± 0.51

*Continuous variables are shown as means ± SD for a normal distribution or medians (interquartile range) for a non-normal distribution. HD, hemodialysis; RDI, recommended daily intake; SFA, saturated fatty acids; MUFA, monounsaturated fatty acids; PUFA, polyunsaturated fatty acids; Cu, copper; Zn, zinc.*

* p < 0.05,

** *p < 0.01,*

*** *p < 0.001 inadequate Zn intake vs. adequate Zn intake*.

Dietary intake data of HD patients is presented in Table 1. Considering the estimated dietary intake of PUFA, the nutritional n-6/n-3 ratio was notably high among HD patients. The median contribution of PUFA to total energy intake was 6.84 ± 3.28%. Further data analyses indicated higher n-3 PUFA intake among HD patients with adequate zinc intake in comparison with those whose intake was below the recommendations: 0.69 (0.56–0.94) vs. 0.53 (0.39–0.69) g/day. This difference reach statistical significance (*p* = 0.028). There were no differences for n-6, n-6/n-3, total PUFA and copper intake comparing these groups (Table 1). Group comparison based on zinc intake adequacy revealed that HD patients with appropriate dietary zinc intake had higher caloric intake (*p* = 0.03), protein (*p* = 0.003), SFA (*p* = 0.005), and MUFA (*p* = 0.012).

To acknowledge the impact of diverse dietary sources on zinc bioavailability and subsequent status, the contribution of various food groups to total zinc intake is presented in Figure 1A. In this study, the main dietary source of zinc were food items belonging to meat and meat product group with mean contribution of 3.26 mg/day, representing 34.72% of the total estimated dietary intake of zinc, so we presented the distribution of zinc daily intake from foods from meat and meat products group in Figure 1B. In this food group, a dominant source was beef meat, followed by pork and chicken meat. Based on food consumption analysis, other major sources were grains and grain products, miscellaneous food products, as well as milk, milk products and substitutes with 14.03, 13.77 and 10.39% contribution, respectively. In the miscellaneous product group important zinc dietary sources were bakery yeast and seasonings such as powdered red paprika, dried parsley leaves, ground nutmeg, and powdered garlic. Only 2.66% of total zinc intake was attributed to seafood and related products.

### Correlations of Biochemical and Anthropometric Parameters With Zinc Intake and Status

**Figure 2:**
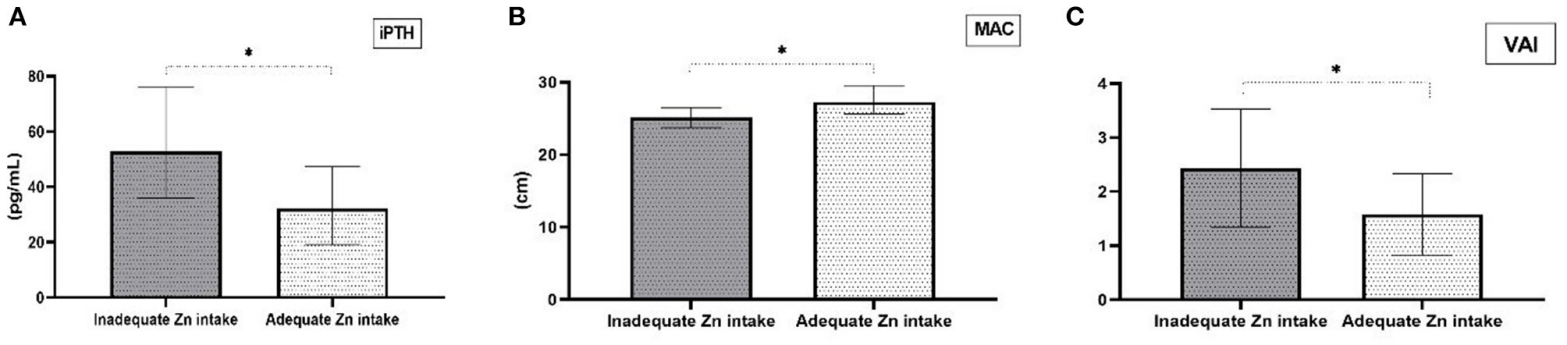
Differences in clinical and anthropometric parameters comparing groups with adequate and inadequate dietary Zn intake. **(A)** Median values for intact parathyroid hormone (iPTH) levels (1st-3^rd^ interquartile for inadequate Zn intake = 36–75.9; 1st-3rd interquartile for adequate Zn intake = 19–47.3), **(B)** Median values for mid-arm circumference (MAC) (1st-3rd interquartile for inadequate Zn intake =23.7–26.5; 1st-3rd interquartile for adequate Zn intake = 25.6–29.55), and **(C)** Mean ± standard deviation for visceral adiposity index (VAI). *p < 0.05 for inadequate Zn intake vs adequate Zn intake.

Group comparison based on zinc intake adequacy revealed that HD patients with appropriate dietary zinc intake had lower iPTH concentrations (*p* = 0.047), VAI values (*p* = 0.020) and higher MAC (*p* = 0.031) (Figures 2A–C). There were no other statistically significant differences in biochemical and anthropometric parameters between these two groups. Moreover, further analysis of obtained data showed that there are significant correlations between dietary zinc intake and iPTH, VAI and MAC values (Table 2). The only significant difference in the fatty acid profile in HD patients with inadequate zinc intake was a lower level of n-3 PUFA compared to HD patients with adequate zinc intake (Table 3). Nevertheless, low EPA+DHA values and high n-6/n-3 ratio were present in both groups.

**Table 2:** Characteristic of the hemodialysis patients.

**Variable**	**All patients**	**Adequate Zn intake**	**Inadequate Zn intake**
Hg (g/L)	101.0 ± 15.2	102.8 ± 11.8	102.6 ± 18.3
Urea (mmol/L)	23.9 ± 4.8	24.1 ± 5.8	23.3 ± 4.7
Creatinine (μmol/L)	913 ± 147	967 ± 135	876 ± 161
Kt/V	1.47 ± 0.32	1.34 ± 0.27	1.49 ± 0.37
Protein (g/l)	67.5 (63–70)	68 (66.5–69)	67.5 (63–70)
Albumin (g/l)	37.5 (36–40)	37 (35–39)	38 (37–40)
Total cholesterol (mmol/L)	4.52 ± 0.92	4.73 ± 0.92	4.40 ± 1.35
LDL cholesterol (mmol/L)	3.09 ± 0.78	2.95 ± 0.82	3.19 ± 0.93
HDL cholesterol (mmol/L)	F: 0.995 (0.91–1.08)	F: 0.955 (0.91–1)	F: 1.41 (1.09–1.46)
	M: 1.065 (0.90–1.29)	M: 1.185 (0.98–1.4)	M: 0.9 (0.64–1.14)
Triglycerides (mmol/L)	1.21 (1.025–1.575)	1.11 (1.02–1.51)	1.50 (1.00–1.93)
Tg/HDL	1.28 (0.91–1.69)	1.10 ± 0.48	1.52 ± 0.73
CRP ≤ 3 mg/L n (%)	12 (42%)	6/12	6/16
CRP > 3 mg/L n (%)	28 (58%)		
WC (cm)	F: 82.1 ± 10.8	F: 78.5 ± 11.3	F: 87.1 ±8.8
	M: 95.6 ± 11.4	M: 96.8 ±13.3	M: 94.5 ±9.5
% Body fat	F: 30.6 ± 10.8	F: 28.2 ± 2.3	F: 29.1 ±5.3
	M: 25.5 ± 8.4	M: 23.7 ±9.4	M: 26.1 ± 8.0
MAC (cm)	25.6 ± 3.8	27.25 (25.6–29.55)	25.1 (23.7–26.5)^*^
VFA (cm^2^)	96.2 ± 44.6	93.2 ± 55.	102.7 ± 32.2
VAI	2.00 ± 1.01	1.58 ± 0.75	2.44 ± 1.09^*^
Iron (μmol/L)	14.8 ± 5.3	13.8 ± 4.4	15.2 ± 6.1
iPTH (pg/ml)	43.45 (19.4–64.7)	32.3 (19–47.3)	52.8 (36–75.6)^*^
Vitamin D (nmol/L)	35.87 ± 9.81	35.46 ± 11.46	34.94 ± 8.03

*Continuous variables are shown as means ± standard deviation for a normal distribution or medians (interquartile range) for a non-normal distribution. BMI, body mass index; F, female; M, male; CRP, C-reactive protein; Hg, hemoglobin; Tg, Triglyceride; WC, waist circumference; MAC, mid arm circumference; VFA, visceral fat area; VAI, visceral adiposity index; iPTH, intact parathyroid hormone.*

* *p < 0.05 inadequate Zn intake vs adequate Zn intake, #distribution of data could not be tested as there are less than 5 data (n = 3)*.

**Table 3:** Plasma fatty acid status in hemodialysis patients.

**Fatty acid**	**All patients**	**Adequate Zn intake**	**Inadequate Zn intake**
**%**		***n* = 18**	***n* = 22**
16:0	28.60 ± 1.94	28.44 ± 2.03	28.31 ± 2.28
18:0	19.06 (17.68–19.81)	17.80 (17.04–19.70–24.82)	19.36 (18.36–20.29)
**SFA**	47.52 ± 2.32	46.85 ± 2.22	47.96 ± 2.30
16:1n−7	0.38 (0.26–0.46)	0.36 (0.22–0.45)	0.40 (0.27–0.48)
18.1n-9	9.61 (8.54–10.25)	9.73 (8.38–10.19)	9.87 (8.86–10.44)
18:1n−7	1.85 (1.70–2.09)	1.73 (1.66–2.02)	1.95 (1.74–2.08)
**MUFA**	11.84 (11.03–12.68)	11.81 (10.72–12.20)	12.31 (11.22–12.87)
18:2n-6 (LA)	21.89 ± 3.14	22.22 ± 2.93	21.64 ± 3.99
20:3n-6 (DGLA)	2.87 ± 0.66	3.02 ± 0.74	2.94 ± 0.63
20:4n-6 (AA)	11.78 ± 2.25	12.14 ± 2.06	11.61 ± 2.62
22:4n-6	0.66 (0.50–0.90)	0.67 (0.46–1.01)	0.60 (0.45–0.71)
**n-6**	37.27 ± 3.03	38.12 ± 2.69	36.79 ± 7.48
18:3n-3	0.11 (0.08–0.16)	0.12 (0.08–0.15)	0.09 (0.07–0.19)
20:5n−3 (EPA)	0.22 (0.16–0.31)	0.24 (0.14–0.32)	0.18 (0.16–0.32)
22:5n-3(DPAn-3)	0.56 (0.48–0.69)	0.62 (0.48–0.80)	0.54 (0.44–0.59)
22:6n-3 (DHA)	2.26 ± 0.57	2.36 ± 0.62	2.08 ± 0.41
**n-3**	3.25 ± 0.74	3.42 ± 0.83	2.97 ± 0.51^*^
**n-6/n-3**	11.99 ± 2.54	11.71 ± 2.66	12.68 ± 2.20
EPA+DHA	2.51 ± 0.60	2.61 ± 0.63	2.29 ± 0.42
PUFA	40.52 ± 3.31	41.54 ± 2.71	39.77 ± 3.61
**D5D**	4.28 ± 1.15	4.20 ± 1.09	4.06 ± 1.11
**D6D**	0.128 (0.106–0.153)	0.129 (0.109–0.149)	0.127 (0.102–0.185)
**SCD16**	0.013 (0.009–0.016)	0.012 (0.008–0.016)	0.014 (0.010–0.016)
**SCD18**	0.49 (0.43–0.57)	0.53 (0.45–0.57)	0.49 (0.44–0.57)
LA/DGLA	8.10 ± 2.53	7.80 ± 2.17	7.79 ± 2.72
ELO	2.47 (0.96–11.0)	3.29 (3.10–11.00)	2.51 (1.00–5.17)

*Continuous variables are shown as means ± standard deviation for a normal distribution or medians (interquartile range) for a non-normal distribution; SFA, saturated fatty acids; MUFA, monounsaturated fatty acids; LA, linoleic acid; DGLA, dihomo-γ-linolenic acid; AA, arachidonic acid; EPA, eicosapentaenoic acid; DPA, docosapentaenoic acid; DHA, docosahexaenoic acid; PUFA, polyunsaturated fatty acids; D5D, Δ-5-desaturase; AA/DGLA (20:4 n-6/20:3 n-6), D6D, Δ-6-desaturase: DGLA/LA (20:3 n-6/18:2 n-6); SCD16 and 18, stearoil-CoA desaturase 16 and 18, ELO, elongase: (DPAn-3/EPA),*

* *p < 0.05 inadequate Zn intake vs. adequate Zn intake*.

Table 4 represents the associations of biochemical and anthropometrics parameters with dietary intake of zinc, serum zinc and copper status. Dietary zinc intake did not correlate with serum zinc concentration in HD patients (data not shown). The inverse associations were found between zinc intake estimates and LDL cholesterol (*r* = −0.332, *p* = 0.036) as well as iPTH levels (*r* = −0.317, *p* = 0.046) (Table 4). Dietary intake of zinc was directly associated with the anthropometric parameter MAC and inversely with VAI (*p* < 0.05, for both parameters). There were no significant correlations between the serum zinc concentrations and analyzed biochemical parameters. However, significant inverse correlations between the serum Cu/Zn ratio and serum albumin (r = −0.404, *p* = 0.009), iron (r = −0.351, *p* = 0.026), PTH (*r* = −0.332, *p* = 0.036) concentration and Kt/V (*r* = −0.317, *p* = 0.046) were noted. In addition, the serum Cu/Zn ratio was directly associated with HD period (*r* = 0.355, *p* = 0.023), CRP (*r* = 0.315, *p* = 0.048), BMI (*r* = 0,384, *p*= 0.014) and VFA (r = 0.327, *p* = 0.040). Serum copper concentration directly correlated with BMI (r = 0.384, *p* = 0.014) and VFA (*r* = 0.361, *p* = 0.023) and inversely correlated with serum albumin concentration (*r* = −0.449 *p* = 0.004).

**Table 4:** Correlation between zinc intake and status values with the different biochemical parameters in hemodialysis patients.

	**Hg**	**Iron**	**CRP**	**HD ** **Per**	**Kt/V**	**Urea**	**sCr**	**Alb**	**TC**	**LDL**	**Tg**	**Tg / ** **HDL**	**iPTH**	**Vit D**	**BMI**	**MAC**	**VFA**	**VAI**
**Dietary**																		
**Zn**																		
**r**	*0.021*	*−0.078*	*−0.140*	*−0.168*	*−0.132*	*−0.120*	*0.222*	*0.202*	*0.051*	*−0.332*	*−0.039*	*−0.168*	*−0.317*	*0.087*	*0.005*	*0.449*	*0.088*	*−0.317*
**p**	*0.899*	*0.629*	*0.388*	*0.300*	*0.415*	*0.460*	*0.170*	*0.211*	*0.750*	*0.036*	*0.809*	*0.300*	*0.046*	*0.589*	*0.977*	*0.004*	*0.589*	*0.046*
**Serum**																		
**Zn**																		
**r**	*0.113*	*0.021*	*0.009*	*−0.089*	*0.132*	*−0.118*	*0.032*	*0.118*	*−0.039*	*0.051*	*0.083*	*0.001*	*0.113*	*0.021*	*0.083*	*0.010*	*0.113*	*0.069*
**p**	*0.486*	*0.900*	*0.955*	*0.582*	*0.415*	*0.469*	*0.842*	*0.470*	*0.809*	*0.753*	*0.611*	*0.998*	*0.485*	*0.896*	*0.611*	*0.925*	*0.485*	*0.669*
**Serum**																		
**Cu**																		
**r**	*0.118*	*−0.214*	*0.231*	*0.010*	*−0.256*	*−0.032*	*0.118*	*−0.449*	*0.056*	*−0.064*	*0.010*	*−0.021*	*−0.177*	*−0.038*	*0.383*	*0.185*	*0.361*	*0.094*
**p**	*0.470*	*0.183*	*0.151*	*0.950*	*0.111*	*0.842*	*0.470*	*0.004*	*0.729*	*0.696*	*0.950*	*0.900*	*0.273*	*0.815*	*0.015*	*0.252*	*0.023*	*0.560*
**Cu/Zn**																		
**r**	*0.118*	*−0.214*	*0.231*	*0.010*	*−0.256*	*−0.032*	*0.118*	*−0.449*	*0.056*	*−0.064*	*0.010*	*−0.021*	*−0.177*	*−0.038*	*0.383*	*0.185*	*0.361*	*0.094*
**p**	*0.415*	*0.026*	*0.048*	*0.023*	*0.046*	*0.454*	*0.879*	*0.009*	*0.683*	*0.522*	*0.539*	*0.751*	*0.029*	*0.720*	*0.016*	*0.155*	*0.040*	*0.770*

*Alb, albumin; sCr, serum creatinine; HD per, hemodialysis period; TC, total cholesterol*.

### Correlations of PUFA and Estimates of Desaturase Activity With Zinc Status

As shown in Table 5, no statistically significant correlations were observed between dietary zinc intake and plasma phospholipids' levels of individual n-6 and n-3 PUFAs neither with estimated desaturase activities among HD patients. Positive correlations were found for serum zinc concentration with DGLA (*r* = 0.357, *p* = 0.024) and D6D (*r* = 0.311, *p* = 0.037) and inverse associations with the DGLA/LA ratio (*r* = −0.335, *p* = 0.033). Serum copper concentration was inversely associated with EPA (*r* = −0.465, *p* = 0.03). The Cu/Zn ratio and serum copper concentration directly correlated with ELO (*r* = 0.384, *p* = 0.01 and *r* = −0.575, *p* < 0.001, respectively).

**Table 5:** Correlation between Zinc intake and status values with plasma PUFA profile and estimated desaturase activity in hemodialysis patients.

	**LA**	**ALA**	**DGLA**	**AA**	**EPA**	**DHA**	**n-6/n-3**	**EPA +** **DHA **	**D5D**	**D6D**	**LA /** **DGLA**	**SCD16**	**SCD18**	**ELO**
**r**	*0.173*	*0.213*	*0.133*	*0.023*	*0.026*	*−0.048*	*0.007*	*0.058*	*−0.092*	*0.011*	*−0.025*	*−0.036*	*0.054*	*0.073*
**p**	*0.284*	*0.182*	*0.406*	*0.892*	*0.874*	*0.742*	*0.955*	*0.729*	*0.564*	*0.947*	*0.883*	*0.827*	*0.761*	*0.668*
**Serum**														
**Zn**														
**r**	*−0.125*	*−0.218*	*0.357*	*0.250*	*−0.292*	*−0.052*	*0.165*	*−0.112*	*−0.168*	*0.331*	*−0.335*	*−0.229*	*−0.183*	*0.220*
**p**	*0.442*	*0.174*	*0.024*	*0.118*	*0.064*	*0.748*	*0.307*	*0.491*	*0.296*	*0.037*	*0.033*	*0.155*	*0.277*	*0.168*
**Serum**														
**Cu**														
**r**	*−0.032*	*−0.147*	*0.256*	*0.089*	*−0.465*	*0.079*	*0.060*	*−0.027*	*−0.215*	*0.181*	*−0.202*	*0.165*	*−0.202*	*0.575*
**p**	*0.842*	*0.365*	*0.111*	*0.583*	*0.003*	*0.627*	*0.704*	*0.868*	*0.184*	*0.263*	*0.211*	*0.310*	*0.211*	* <0.001*
**Cu/Zn**														
**r**	*0.118*	*0.021*	*−0.023*	*−0.118*	*−0.364*	*0.093*	*−0.060*	*0.036*	*−0.087*	*−0.070*	*0.063*	*0.035*	*−0.027*	*0.384*
**p**	*0.470*	*0.899*	*0.907*	*0.470*	*0.090*	*0.566*	*0.715*	*0.827*	*0.589*	*0.666*	*0.697*	*0.829*	*0.867*	*0.014*

*LA, linoleic acid; DGLA, dihomo-γ-linolenic acid; AA, arachidonic acid; ALA, alfa-linolenic acid; EPA, eicosapentaenoic acid, DHA, docosahexaenoic acid; D5D, Δ-5-desaturase; AA/DGLA (20:4 n-6/20:3 n-6); D6D, Δ-6-desaturase; DGLA/LA (20:3 n-6/18:2 n-6); SCD16 and 18, stearoil-CoA desaturase 16 and 18; ELO, (DPAn-3/EPA)*.

## Discussion

To the best of our knowledge, this is the first study evaluating zinc status in hemodialysis patients, considering dietary zinc intake, serum zinc levels and mutual relations with fatty acid profile, and estimated desaturase activities. Among studied patients, both zinc deficiency and altered Cu/Zn ratio were observed. Inadequate dietary zinc intake was present up to 55% of hemodialysis patients and there was no significant correlation between the estimated intake and serum zinc concentration. Based on our findings, zinc dietary intake did not correlate with serum PUFA status and estimated desaturase activity in hemodialysis patients. Furthermore, data revealed the absence of association between serum zinc concentration and analyzed biochemical parameters. It is noteworthy that all the studied hemodialysis patients had zinc deficiency, determined according to low serum zinc concentration as known as a sensitive biomarker of zinc status. This led to no statistically significant difference in the zinc levels and Cu/Zn ratio compared hemodialyzed patients with adequate and inadequate zinc intake. Results of this study indicate that the serum zinc and copper concentrations and their ratio were related to modified fatty acid PUFA profile in hemodialysis patients, with DGLA, EPA, LA/DGLA and estimated D6D activity.

Our results are in accordance with previously published data suggesting that hemodialysis patients have low serum zinc concentration (5, 19–23). As reported in the present study, dietary zinc intake is quite often not correlated with plasma/serum zinc status (37, 51). Fifty-five percent of recruited patients had zinc intake below the recommended level for patients with CKD and all had zinc deficiency (defined as serum concentration below 60 μg/dL). Severe zinc deficiency can manifest clinically when its serum values decrease to 40 μg/dL. Mean serum zinc concentration of 38.8 ± 7.72 μg/dL obtained in these samples could be considered as indicative for zinc supplementation. Although only 1% of total zinc in the body is present in circulation, serum zinc is still regarded as an important biomarker and useful indicator of dietary zinc restriction, zinc supplementation, and clinical deficiency. The recently determined interval for serum zinc concentration in the adult Serbian population (from 40 to 82,5 μg/ dL) strongly implies that even our healthy population could be at risk to develop zinc deficiency (52). Therefore, adequate oral zinc supplementation and/or foodstuff fortification should be considered to prevent the deleterious effects caused by zinc deficiency (53). It is important to notice that meta-analysis by Wang et al. (54) showed that zinc supplementation with median dose of 45 mg/day and duration of median 60 days results in higher serum zinc levels and lower CRP and malondialdehyde levels in hemodialyzed patients. Thus, evidence supported that zinc supplementation in doses that are much higher than recommended values for dietary intake (8–12 mg/day for women, 10–15mg/day for men) may improve serum zinc levels in hemodialysis patients. Increasing number of CKD patients is considered a major public health problem and there is the new evidence that low dietary zinc intake may increase the risk of CKD development in individuals with normal renal function (55). Low serum zinc concentrations among hemodialysis patients are supposed to result from zinc removal during the treatment itself, decreased albumin levels, diminished gastrointestinal zinc absorption, and antagonistic effects of copper to their common carrier – metalothionine, due to its higher affinity to copper compared to zinc (16, 56). In addition, a high intake of copper, iron or phytic acid could cause malabsorption of dietary zinc (57). The molar ratio of phytate and zinc has been shown to be a predictor of zinc absorption; however, data for dietary amounts of phytic acids are still scarcely available in food composition databases. A nutrition plan targeting plant-based food was not recommended to studied hemodialysis patients due to the high content of potassium and phosphates. Given that the major contributor to zinc intake were meat and meat products, participants of the present study dominantly obtained zinc from high bioavailability sources. Group comparison based on zinc intake adequacy revealed that our hemodialysis patients with appropriate dietary zinc intake had daily higher energy, protein, total fats and SFA, MUFA and n-3 PUFA consumption.

It is assumed that disruption of zinc and copper levels could be the cause of clinical deterioration as well as negative outcomes in hemodialysis patients. Previous findings indicated that the increased plasma Cu/Zn ratio reflects the disturbance in zinc and copper homeostasis and could be applied as a clinical predictor for patients' inflammatory status (10). It is suggested that the optimum serum Cu/Zn-ratio lies between 0.7 and 1.0 (58). Participants in the present study exhibited abnormally increased Cu/Zn-ratio with a mean value of 2.76 ± 0.68. Trendafilov et al. (56) found a negative correlation between zinc status and markers of inflammation (CRP and IL-6), and a direct association of these parameters with copper status. Given the association found with the CRP as an indicator of the inflammatory response, clinical significance of Cu/Zn ratio for treatment outcome and further progression of renal failure is evident in our study population.

According to the European Society for Clinical Nutrition and Metabolism guideline for hospitalized patients with acute or CKD, zinc and copper levels should be measured for nutrition screening and supplemented accordingly (59). There is evidence implying that serum zinc concentration is reduced by hypoalbuminemia in CKD patients, as zinc is bound to albumin in the circulation (60). Our study showed that the Cu/Zn ratio is inversely correlated with albumin levels. This study did not identify such associations between serum zinc concentration and explored biochemical parameters. This might be due to the limited sample size, which resulted in low statistical power for detecting outcomes of the serum zinc variable. The Cu/Zn ratio has been recognized as a better indicator of the significance of zinc deficiency than the concentration of zinc and copper in serum separately. In the present study inverse association between the serum Cu/Zn ratio and serum iron and iPTH concentration was observed among hemodialysis patients.

Zinc deficiency is suspected to cause an increase in PTH due to its contribution in maintaining calcium homeostasis (61). Previously published studies showed a significant negative correlation between serum levels of iPTH and zinc in hemodialyzed children (62), but this correlation was not highlighted for the adult patient population (63). Based on our findings, estimated dietary zinc intake and Cu/Zn ratio were inversely associated with serum concentrations of iPTH, but there was no significant association between serum zinc levels and iPTH levels. Taken all together, our results indicate that zinc intake and its status together with Cu/Zn balance in serum are associated with iPTH levels and consequently calcium homeostasis. The mechanism of PTH effects on plasma zinc concentration in CKD was investigated by Chen et al. (64) who found that PTH enhanced extra renal zinc disposal and increased zinc uptake by liver, suggesting that the over-secretion of PTH could be present in zinc deficiency. Furthermore, low vitamin D status leads to reduced efficiency of intestinal calcium absorption, and the body reacts by increasing the secretion PTH. Prescription of activated vitamin D to a CKD patient with high PTH levels is, therefore, a justified clinical solution. It is noteworthy that all the studied patients in our sample had vitamin D deficiency. Nevertheless, serum vitamin D levels did not correlate with zinc status.

Previously published data revealed that zinc supplementation could significantly reduce total cholesterol, LDL-cholesterol, and triglycerides in the general population (63). However, a recent meta-analysis showed no effect on the lipid profile of zinc supplementation in patients subjected to maintenance hemodialysis (54). A statistically significant inverse association was found between LDL-cholesterol levels and estimated dietary zinc intake, but no significant relationships were found with serum zinc concentration and Cu/Zn ratio. We obtained significant differences for iPTH, VAI, and MAC between the groups with adequate and inadequate dietary zinc intake, and its association with these parameters. However, we did not obtain correlations of iPTH, VAI, and MAC with serum zinc concentration. This could indicate the influence of deviations of zinc homeostasis and redistribution in tissue and cell (e.g. zinc concentration in erythrocytes). Some authors declare that it is not clear yet whether low serum/plasma zinc levels in CKD patients represent genuine deficiency or its low levels are due to redistribution in different biological compartments (65). Recently, serum zinc levels were found to positively correlate with the nutritional status assessed by measuring abdominal fat area using computed tomography in patients with advanced CKD undergoing hemodialysis (66). In our study, no statistically significant correlations were determined between the serum zinc level concentration and anthropometric measurements. Nevertheless, based on our observations, the Cu/Zn ratio positively correlated with BMI and VFA. Furthermore, the results of our study indicated that dietary zinc intake directly correlated with the MAC and inversely with VAI, a reliable indicator of visceral fat function associated with cardiometabolic risk. There is substantial evidence that higher BMIs are associated with an increased risk of CVD and mortality in the general population, but a reverse relationship is observed in HD patients and this phenomenon is known as the “risk factor paradox” (67). It is, however, unclear whether the survival advantage associated with higher BMIs among hemodialysis patients is caused by increased muscle mass, fat mass, or both. Previous findings indicated that zinc supplementation increase body weight and BMI in hemodialysis patients (68, 69).

Blood fatty acid composition seems to be an independent risk factor for CVD in patients subjected to HD (34, 70–72). It reflects both nutritional intake and endogenous synthesis of fatty acids, but there is still not much available information on dietary fatty acid intake in patients undergoing hemodialysis. In a recent study, Khor et al. (73) reported suboptimal dietary intake of PUFAs (<5% of total calories). The American Heart Association recommends replacing saturated fatty acids with PUFAs (>10% of total calories) for the general population to reduce the risk for CVD (74). Inadequate intake of total PUFAs was found in 50% of hemodialysis patients in this study. The mean dietary intake of fish and seafood was 10.5 g/day i.e. 73.5 g/week. These findings suggest suboptimal intake of n-3 PUFA as well, since two servings of fish (180g) a week is recommended (75). Based on Wijendran et al. (76), if the consummation of LA, ALA, EPA+DHA were in accordance with the recommendations, the dietary n-6/n-3 ratio should be approximately 6. In the present study 33 of 40 participants had a higher dietary n-6/n-3 PUFA ratio. Therefore, dietary habits of studied hemodialysis patients indicate the need for better nutritional strategies to achieve adequate fatty acid status. However, adequate zinc intake in our study population was accompanied with significantly higher intake of energy, total fats, SFA, MUFA and protein. According to results of our study red meat represented the main dietary source of zinc. Red meat represents a rich dietary source of zinc and proteins but also other nutrients such as SFA and cholesterol, limiting of its consumption is proposed strategy to reduce CVD risk in CKD patients, making even more difficult to reach adequate zinc intake by diet (77). Currently, there is no consensus in nutrition guidelines for the optimal dietary fatty acid intake targeting specifically CKD patients, but high dietary intake of zinc by consumption of foods abundant in zinc may lead to overconsumption of SFA. According to results of this study the zinc supplementation in CKD patients on hemodialysis, may be necessary to improve serum zinc status as the patients with adequate dietary zinc intake are shown to be zinc deficient, but could be a good strategy to ensure adequate zinc intake without high intake of some other nutrients such as SFA. Due to the recommended restriction of animal protein intake in patients with CKD supplementation is indicative for resolving zinc deficit in patients on hemodialysis.

A recent systematic review regarding the blood fatty acid status in dialysis patients from different countries revealed that the reported diversity of n-3 PUFA levels could be attributed to the variety of dietary habits (78). Among participants in this study, very low levels of EPA+DHA were observed in serum phospholipids. These findings are not unexpected since the dietary intake of fish and seafood was significantly below the recommendations. Our previous studies have already demonstrated markedly low n-3 PUFA status in dialyses population as well as its association with other clinical parameters, especially inflammation markers (29, 47, 79). This current study indicates that besides low n-3 PUFA status their dietary intake is also inadequate and accompanied by high n-6/n-3 ratio.

Essential fatty acids LA and ALA cannot be synthesized in humans and must be provided by the diet. However, they can be metabolized to long-chain PUFAs. DGLA, AA, and 22:4n-6 are the major products of LA desaturation/elongation metabolic pathway, while the ALA can be converted to EPA, DPAn-3, and DHA. Although these conversions seem to be relatively inefficient in humans, our previous research suggested beneficial effects of administration of ALA-rich seed mixture for improving EPA and DHA levels in hemodialysis patients (47). In fact, in the recent study authors hypothesized that uremic milieu found in hemodialysis patients could affect the n-3 PUFA metabolic pathway (73). Previous investigations have shown that serum/plasma zinc and copper levels could be associated with LA/DGLA values in healthy, dyslipidemic obese subjects, and children (35, 37, 80). To our best knowledge, there is no literature data regarding the relationship between zinc and copper status with estimates of desaturase activities and relative abundance of individual PUFA in HD population.

In the present study, zinc status was low in all patients undergoing hemodialysis, but the finding that it is directly associated with D6D is not unexpected. The inverse association between serum zinc status and LA/DGLA ratio has been determined in healthy subjects and dyslipidemic obese adult subjects, as well as children (35, 37, 80). Therefore, LA/DGLA was proposed as an emerging candidate biomarker for zinc status assessment (37). The obtained statistically significant inverse relationship between serum zinc concentrations and LA/DGLA confirms sensitivity of this biomarker in zinc deficient population. In line with other studies, we also found that DGLA levels were positively correlated with zinc status (35, 37). This fatty acid can be metabolized to one series of prostaglandins which possess anti-inflammatory properties, unlike two series of prostaglandins produced from AA generally viewed as pro-inflammatory (69). AA/DGLA ratio which is generally elevated in chronic renal patients undergoing HD is regarded as an important predictor of poor clinical outcomes (72). In this study, copper levels correlated inversely with EPA levels and directly with estimates of ELO activity, while the Cu/Zn ratio directly correlated with ELO activity and was not associated with EPA levels. These findings could be important as there is no literature data for copper status association with EPA levels and estimated ELO activity and scientific evidence suggests that EPA supplementation could decrease the all-cause mortality in patients undergoing HD treatment (71).

Based on a contrite meta-analysis of early and preliminarily available data, CKD seems to be associated with enhanced risk of severe COVID-19 infection. Multiple aspects of the immune system are affected by zinc. The prevalence of CKD in COVID-19 patients has been reported to be about 0.09–47.1% (81). Novel data suggest that oral zinc supplementation (80 mg/day of elemental zinc) administered at the first signs and symptoms of COVID-19 could be a promising therapeutic strategy (82). In general, a large body of evidence shows that up to one year of zinc supplementation in doses of 220 mg/d to 660 mg/d in chelated form (corresponding to approximately 50 mg to 150 mg of elemental zinc) seems to be a safe regimen for patients with CKD (54). Recently published studies demonstrated that the administration of oral zinc acetate could increase a risk for copper deficiency in hypoalbuminemic hemodialysis patients (83). A personal nutrition therapeutic approach is necessary for patients with CKD and hemodialysis sessions represent a valuable opportunity to monitor nutritional status and prescribe the appropriate interventions (84–86).

## Limitations

Several limitations of this study should be acknowledged. First, the sample size was relatively small, and our findings should be confirmed and clarified by further larger-scale studies. All involved hemodialyzed patients were zinc deficient, so it was not possible to divide them into groups according to serum zinc levels. Second, dietary intake of zinc, copper and fatty acids were estimated using self-reported, retrospective 24 h nutritional surveys. All methods for the assessment of dietary exposure have their inherent limitations and 24 h surveys may not be the best nutritional tool to estimate the consumption of foods that are not consumed on a daily basis, such as fish, seafood, and seafood products. Finally, statistical analysis was not performed with sex-specific considerations since female participants were underrepresented with only 20% in the studied patient group. Zinc deficiency could cause changes in a plethora of biochemical and clinical parameters that reflect renal function, the efficiency of hemodialysis treatment, dyslipidemia, calcium homeostasis (iPTH and vitamin D), body composition, inflammatory status, and anemia. In addition to factors explored in this study, there are other important parameters such as glucose homeostasis, redox status, and immune function that are relevant for patients undergoing HD, and further research is needed to provide a better understanding of their relationships with zinc status.

## Conclusion

In conclusion, due to the observed zinc deficiency in hemodialysis patients, it is necessary to determine serum zinc levels together with the Cu/Zn ratio as a standard in screening nutritional status. These indices could play an important role in desaturation/elongation of PUFA in this patient population group. Our results underline the need for zinc supplementation in patients undergoing hemodialysis as all patients had suboptimal serum status of zinc including those with adequate dietary intake. Zinc supplementation with high doses that cannot be provided by diet in hemodialysis patients in combination with n-3 PUFA supplements potentially could induce even more favorable change in fatty acids profiles than n-3 PUFAs treatments alone. Importantly, zinc deficiency with an altered n-3 fatty acid profile can affect disease progression and associated with various risk for CVD. More interventional studies are warranted to confirm the benefits of zinc and PUFA supplementation in hemodialysis patients for the prevention and attenuation of adverse health outcomes.

## Data Availability Statement

The raw data supporting the conclusions of this article will be made available by the authors, without undue reservation.

## Ethics Statement

The studies involving human participants were reviewed and approved by Ethical Review Board of the Military Medical Academy, Belgrade, Serbia. The patients/participants provided their written informed consent to participate in this study.

## Author Contributions

MT contributed reagents/materials/analysis tools and performed the statistical analysis and review. MZ accountable for dietary data analysis, presentation of data, review, and editing. BT and MM data collected and review. AS completed mineral analysis, reviewing, and editing. SR responsible for carrying out the human study, for its design and coordination, and editing. DRM conceptualization/study design, methodology, manuscript preparation, review, and editing. All authors contributed to the article and approved the submitted version.

## Conflict of Interest

The authors declare that the research was conducted in the absence of any commercial or financial relationships that could be construed as a potential conflict of interest.

## Publisher's Note

All claims expressed in this article are solely those of the authors and do not necessarily represent those of their affiliated organizations, or those of the publisher, the editors and the reviewers. Any product that may be evaluated in this article, or claim that may be made by its manufacturer, is not guaranteed or endorsed by the publisher.

## Abbreviations

AA: arachidonic acid
ALA: alfa-linolenic acid
Alb: albumin
BMI: body mass index
CKD: chronic kidney disease
CRP: C-reactive protein
Cu: copper
CVD: cardiovascular disease
D5D: Δ-5-desaturase
D6D: Δ-6-desaturase
DGLA: dihomo-γ-linolenic acid
DHA: docosahexaenoic acid
DPA: docosapentaenoic acid
ELO: elongase
EPA: eicosapentaenoic acid
HD: hemodialysis
Hg: hemoglobin
ICP-MS: inductively coupled plasma mass spectrometry
iPTH: intact parathyroid hormone
LA: linoleic acid
MAC: mid arm circumference
MUFA: monounsaturated fatty acids
PUFA: polyunsaturated fatty acids
RDI: recommended daily intake
sCr: serum creatinine
SFA: saturated fatty acids
Tg: Triglyceride
TC: total cholesterol
VAI: visceral adiposity index
VFA: visceral fat area
WC: waist circumference
Zn: zinc.
